# Effect of a patient decision aid on shared decision making in patients with differentiated thyroid cancer: a randomized controlled trial

**DOI:** 10.1093/oncolo/oyag126

**Published:** 2026-04-07

**Authors:** Anna Koot, Petronella Ottevanger, Rosella Hermens, Annemijn Aarts, Janneke Walraven, Han Bonenkamp, Johannes de Wilt, Grard Nieuwenhuijzen, Alexander van der Veen, Mariel Keemers, Wouter Zandee, Charlotte Blanken, Maarten van Aken, Rogier Donders, Peep Stalmeier, Romana Netea-Maier, COMBO study group, Marieke Snel, Esther Broekman, Liesbeth Jansen, Arianne van Bon, Rachel van Leeuwaarde, Menno Vriens, Iris van den Broek, Jan Paul de Boer, Marie-Jose Pauwels, Willemien Menke van der Houven–van Oordt, Annewieke van den Beld, Marleen Kars, Johanna Nin, Lieke Engelen

**Affiliations:** Science Department IQ Health, Radboud University Medical Center, Nijmegen, 6525 GA, The Netherlands; Department of Internal Medicine, Division of Endocrinology, Radboud University Medical Center, Nijmegen, 6525 GA, The Netherlands; Division of Oncology, Department of Internal Medicine, Radboud University Medical Center, Nijmegen, 6525 GA, The Netherlands; Science Department IQ Health, Radboud University Medical Center, Nijmegen, 6525 GA, The Netherlands; Department of Obstetrics and Gynecology, Amsterdam University Medical Center, Location Amsterdam Medical Center, Amsterdam, 1105 AZ, The Netherlands; Division of Oncology, Department of Internal Medicine, Radboud University Medical Center, Nijmegen, 6525 GA, The Netherlands; Department of Surgical Oncology, Radboud University Medical Center, Nijmegen, 6525 GA, The Netherlands; Department of Surgical Oncology, Radboud University Medical Center, Nijmegen, 6525 GA, The Netherlands; Department of Surgery, Catharina Hospital, Eindhoven, Eindhoven, 5623EJ, The Netherlands; Department of Surgery, Catharina Hospital, Eindhoven, Eindhoven, 5623EJ, The Netherlands; Department of General Surgery, Canisius-Wilhelmina Hospital, Nijmegen, Nijmegen, 6532 SZ, The Netherlands; Department of Endocrinology, University Medical Center Groningen, Groningen, Groningen, 9713 GZ, The Netherlands; Department of Surgery, Rijnstate Hospital, Arnhem, Arnhem, 6815 AD, The Netherlands; Department of Internal Medicine, Haga Hospital, The Hague, Den Haag, 2545 AA, The Netherlands; Science Department IQ Health, Radboud University Medical Center, Nijmegen, 6525 GA, The Netherlands; Science Department IQ Health, Radboud University Medical Center, Nijmegen, 6525 GA, The Netherlands; Department of Internal Medicine, Division of Endocrinology, Radboud University Medical Center, Nijmegen, 6525 GA, The Netherlands

**Keywords:** thyroid cancer, surgery, tyrosine kinase inhibitors, shared decision making, patient decision aids

## Abstract

**Background:**

: Over the last decades optimal treatment for patients with differentiated thyroid cancer (DTC) is debated. Two treatment decisions for patients who could benefit from more individualized approaches and shared decision making (SDM), are the extent of surgery decision in patients with low-risk DTC, and the decision to start or delay the treatment with Tyrosine Kinase Inhibitors (TKIs) in patients with advanced DTC. Our aim is to examine the effect of a Patient Decision Aid (PtDA) on observed SDM, while physicians were trained in SDM.

**Methods:**

In this multicenter RCT (2020-2024), all physicians (*n* = 26) received a 5-hour SDM training. Patients (*n* = 86) with DTC were randomized to receive a PtDA or care as usual. The primary outcome was the observed SDM as rated by blinding observers with the observing patient involvement in decision making (OPTION5) scale from audio-recorded consultations. Secondary outcomes included well-being measures, information-related measures, and decision-related measures.

**Results:**

Mean OPTION5 scores were 52 (range 0-100) for the PtDA and 54 (range 0-100) for the usual care group. The PtDA did not improve observed SDM, nor the secondary outcomes. Results of the Beta testing (*n* = 33) showed that the PtDA was readable (*n* = 30) and helpful for decision making (*n* = 28). All patients recommended using the PtDA.

**Conclusion:**

In this RCT, with high baseline SDM quality provided by trained physicians, PtDAs did not further improve SDM quality. Nevertheless, since all patients recommended the PtDA, future studies should establish the potential benefit of PtDAs, particularly as SDM training is usually not provided.

**Trial registration:**

ClinicalTrials.gov NCT03905369.

Implications for Practice The optimal treatment for differentiated thyroid cancer (DTC) remains a subject of debate. As a result, treatment decisions requires more individualized approaches and shared decision making (SDM). SDM is increasingly promoted, both for ethical reasons and for its positive impact on patient outcomes. In this randomized controlled trial, where baseline levels of SDM were already high, a Patient Decision Aid (PtDA) did not lead to a further improvement in the quality of SDM. Nevertheless, PtDAs may still be valuable, particularly in settings where SDM training is not available.

## Introduction

Most patients with differentiated thyroid cancer (DTC) have a favorable prognosis with an excellent long-term overall survival except for those with advanced radioactive iodine (RAI) refractory disease.^1^ For this reason, clinical practice has shifted towards a more individualized approach, and patients are involved in trade-offs between the harms and benefits of different approaches.^2^ Particularly for patients with low-risk DTC eligible for primary surgery, and patients with advanced RAI refractory DTC, the optimal treatment is debated. As insufficient evidence is available regarding the harms and benefits of treatment in relation to the oncological outcomes and potential overtreatment could unnecessarily affect quality of life (QOL). The American Thyroid Association (ATA) and European Society of Medical Oncology (ESMO) guidelines suggest considering patient preferences, as for some low-risk patients, thyroid lobectomy (TL) alone may be sufficient initial treatment.^3^^,^^4^ Similarly, for patients with advanced RAI refractory disease, the recent European Thyroid Association (ETA) and ESMO guidelines state that the decision about when to start TKIs should include patient preferences with respect to treatment goals, and what patients find important (patient values), regarding benefits and adverse effects of the treatments.^5^ Therefore, shared decision making (SDM) is required.^3^^,^^5^

SDM has been promoted to improve conversations between physicians and their patients and to support patients in making informed decisions that best fit their personal preferences, circumstances, and concerns.^6^ Physicians provide patients with information on existing options and consider patients’ needs, preferences, and values to enable a personalized treatment choice.^7^ In this process, important life goals such as work, sport or family may be involved, and these should be explored together with the patient.^8^^,^^9^ In practice, however, applying SDM in the consultation room and talking about values is difficult. Values are discussed in a minority of decision-making consultations.^7^ Of the SDM skills, exploring patient values is the most difficult one and complicated by different perspectives of physicians and patients.^8^ Moreover, a specific communication training to develop SDM skills was not routinely used and programs vary widely.^10^ In a recent survey conducted within the EORTC Thyroid Carcinoma Group, only 32% of expert physicians reported having received dedicated SDM training.^11^

Our aim is to test three recently developed PtDAs to support abovementioned treatment decisions.^12^ A prospective multicenter randomized controlled trial (RCT), the COMBO (COMmunication BOoster) trial was done, to investigate the added value of these PtDAs on observed SDM in recorded conversations. In general, various SDM training programs for physicians exist, though there is uncertainty whether they are effective.^13^ Only one study investigated the added value of a PtDA to specific communication training on the quality of SDM in patients facing palliative treatment for advanced cancers.^14^ In our trial, all physicians received a dedicated training in SDM to improve the congruence or agreement in values communication between patients and physicians, and to prevent baseline inequalities between the groups. Patients were randomized into two arms, with or without a PtDA. We hypothesized that the PtDAs improve observed SDM. Secondary patient outcomes included well-being measures, information-related measures, and decision-related measures.

## Methods

This paper complies with the SUNDAE checklist for evaluation of PtDA studies^15^ and the Consolidated Standards of Reporting Trials (CONSORT) statement.^16^

### Ethical approvement and informed consent

The COMBO study (COMmunication BOoster) is registered at ClinicalTrials.gov (NCT03905369). The Medical Ethical Committee (METC) of the region Arnhem-Nijmegen approved the study protocol (METC-2018-4521). Patients provided written consent for participation. Due to lagging patient inclusion, the power calculation was reappraised. The initial power calculation was too conservative. The adjusted power calculation was submitted to and approved by our sponsor, Dutch Cancer Society.

### Design

**Figure 1.**
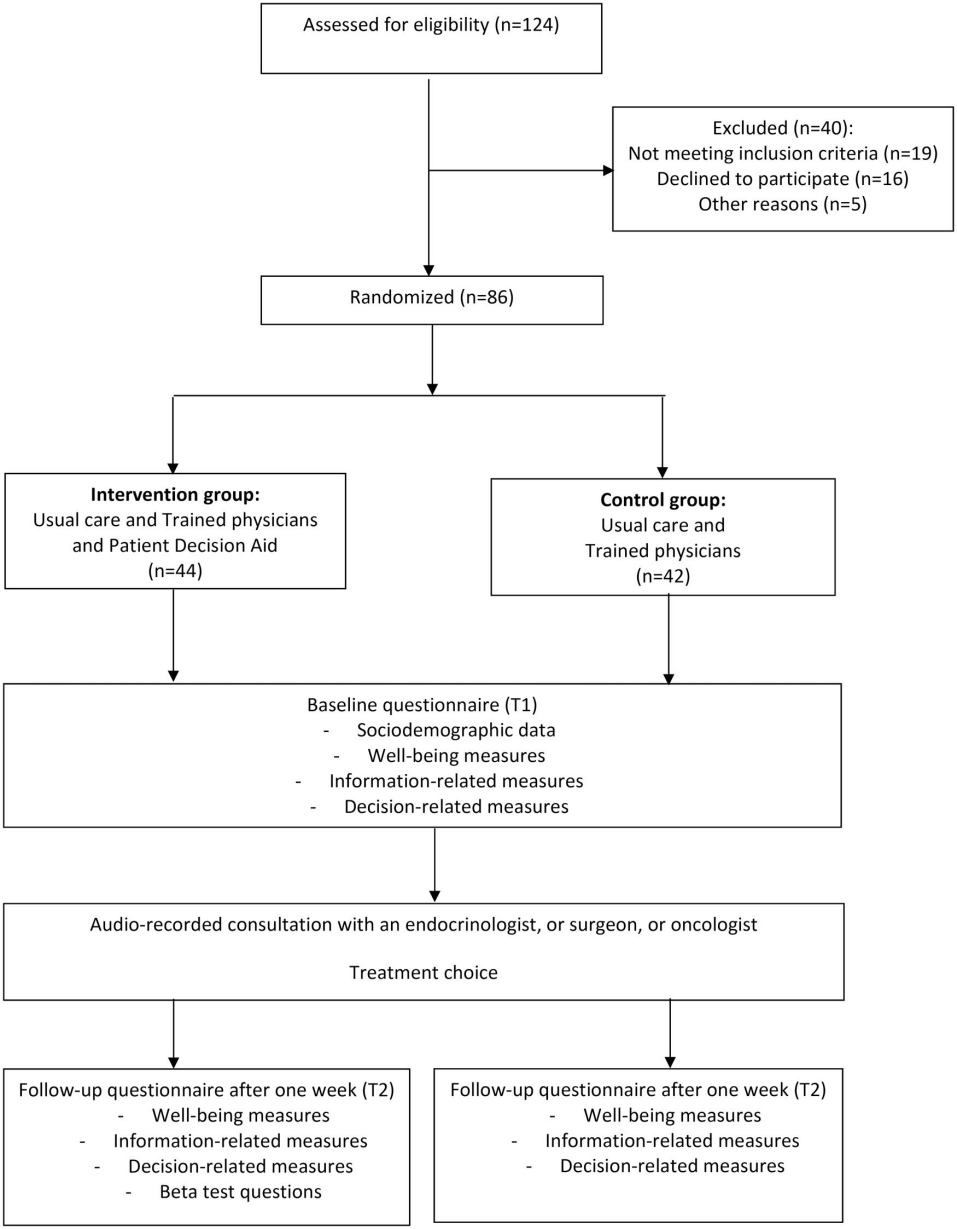
Study design.

Between March 2020 and April 2024, 86 DTC patients, eligible for inclusion, were randomized (1:1) to receive the PtDA (intervention group) or information as usual (control group) (Figure 1). All participating physicians were trained in SDM.

### Setting

Thirteen (of the 25 contacted) hospitals (six academic and seven non-academic) specialized in DTC care in the Netherlands, and the Dutch Thyroid patient association (SchildklierNL) participated. DTC patients are treated in both academic (high- and low-risk patients) and non-academic hospitals (mainly low-risk patients) involving multidisciplinary teams of specialists. The follow-up is carried out by endocrinologists. Patients with advanced RAI refractory disease requiring TKI treatment, are followed-up by oncologists.

### Procedure

Eligible patients were identified by endocrinologists, surgeons, or oncologists and discussed in the local tumor board. Next, the treating physician or nurse informed the patient about the diagnosis, the two available treatment options and trial. After written consent, patients were randomized into two arms, either to receive a PtDA or not before their appointment about the treatment choice. Tumor characteristics were collected from the patients’ medical files. Patients filled out a baseline questionnaire (T1). About one to two weeks later, the SDM consultation took place, which was audio-recorded. Recordings were rated by blinded assessors. Two assessors started double coding a set of ten audio-recordings, and resulting scores were discussed. Inter rater agreement was calculated after each set of ten and considered sufficient if the Intraclass Correlation was higher than .60 reflecting substantial agreement.^17^ When the Inter Rater agreement was insufficient, this process was repeated. After the first set of ten consultations, the Inter Rater agreement was calculated. The intraclass correlation turned out to be sufficient and strong (.81). The remaining recordings were scored independently by two raters without further discussions. Patient outcomes were again assessed with a questionnaire one week after the conversation (T2) (Figure 1).

### Participants

#### Patients

Three groups of patients were eligible: (1) patients with low-risk DTC according to the ATA criteria,^3^ to decide between a TL or TT, (2) patients with low-risk DTC according to the ATA criteria to decide between active surveillance or totalizing thyroidectomy after diagnostic lobectomy, and (3) patients with advanced RAI refractory DTC, to decide between starting or delay starting TKI treatment (either Sorafenib or Lenvatinib, both approved for first line systemic treatment for patients in the Netherlands). Exclusion criteria were contra-indications for surgery or TKI treatment, mental or cognitive problems as assessed by the physician and/or inadequate knowledge of the Dutch language.

#### Physicians

Eight endocrinologists, six oncologists, and twelve surgeons specialized in DTC care and trained in SDM, participated.

### Randomization and blinding

Central randomization took place using a computer-generated randomization list. The same randomization process took place for the whole cohort, regardless the PtDA offered. Participating physicians were blinded to the intervention assignment though the PtDA may have come up in the consultation.

### Interventions

#### Development of the PtDAs

All PtDAs were developed according to the International Patients Decision Aids Standards (IPDAS).^18^ We previously described the development process of the PtDAs used in the present study.^12^ The PtDAs were online, containing general information about DTC, presenting the treatment options, comparing the treatment options, knowledge questions about thyroid cancer and a values clarification exercise, to help patients to choose. The VCE included statements and open-ended questions to explore patients’ needs, preferences and values. Patients were encouraged to share their answers with their physician. The PtDAs developed in this study relate to the situation in the Netherlands and cannot be directly implemented in other countries, because of differences in healthcare practices, clinical guidelines, and specific cultural aspects that influence the needs perceived by patients from different backgrounds. Nonetheless, upon translation and adaptation they can be validated cross-culturally and disseminated in other countries as well.^18^ The hyperlink and translated PtDAs can be found in Supplementary Dataset 1.

#### SDM communication training

The training (5 hours) was based on a model with four essential SDM steps^19^: (1) choice, process and division of roles, (2) inform about the options and pros and cons, (3) explore patients’ needs, preferences and values, (4) make or defer a decision together with the patient. The training started with an e-learning about SDM lasting 30 minutes. Next, physicians received a group training by an experienced trainer lasting three and a half hours. This group training had a special focus on values clarification. The final part of the training, consisted of a 60 minutes individual session with feedback on an audio-recorded consultation, received about 4 weeks after the group training. Additionally, participants received a pocket-size card presenting the four SDM steps with example phrases. The physicians participating in the present study evaluated the training as positive. The training was accredited by the Dutch Association of Internal Medicine (3 CME credits).

### Patient measures

#### Baseline characteristics

Outcome measures are presented in Supplementary Table 1. Characteristics are presented in Table 1.

**Table 1.** Patient and physician characteristics.

Sociodemographics	Decision aid	Usual care	Total
**Patients**			
** Female gender (*n*, %)**	30 (75)	24 (68.6)	54 (72)
** Age, mean (SD)**	55.85 (14.9)	55.51 (16.3)	55.69 (15.5)
** Living with partner (*n*, %)**	31 (77.5)	27 (77.1)	58 (77.3)
** Employed (*n*, %)**	24 (60)	22 (62.9)	46 (61.3)
** Children (*n*, %)**	30 (75)	27 (77.1)	57 (76)
** College education or more (*n*, %)**	16 (40)	11 (31.4)	27 (36)
** Religiously affiliated (*n*, %)**	10 (25)	9 (25.7)	19 (25.3)
**Tumour characteristics**			
** Low-risk DTC (*n*, %)**	36 (81.8)	32 (76.2)	68 (79)
** Diameter nodule on ultrasound (*n*, %)**			67 (77.9)
** 10-20mm (*n*, %)**			24 (27.9)
** 20-30mm (*n*, %)**			28 (32.6)
** 30-40mm (*n*, %)**			15 (17.4)
** Bethesda classification**			67 (77.9)
** Category X (*n*, %)**			4 (4.7)
** Category 0 (*n*, %)**			0 (0)
** Category I (*n*, %)**			2 (2.3)
** Category II (*n*, %)**			1 (1.2)
** Category III (*n*, %)**			3 (3.5)
** Category IV (*n*, %)**			6 (7.0)
** Category V (*n*, %)**			22 (25.6)
** Category VI (*n*, %)**			29 (33.7)
** Histopathology tumor type**			68 (79.1)
** PTC (*n*, %)**			63 (73.3)
** FTC (*n*, %)**			3 (3.5)
** Other (*n*, %)**			2 (2.3)
** TNM stage**			68 (79.1)
** T1 (*n*, %)**			6 (7.0)
** T2 (*n*, %)**			20 (23.3)
** T3 (*n*, %)**			41 (47.7)
** NX (*n*, %)**			3 (3.5)
** N0 (*n*, %)**			65 (75.6)
** MX (*n*, %)**			58 (67.4)
** M0 (*n*, %)**			10 (11.6)
** Advanced RAI refractory DTC**	8 (18.2)	10 (23.8)	18 (21)
** TNM stage (*n*, %)**			18 (21)
** Tx (*n*, %)**			1 (1.2)
** T2 (*n*, %)**			3 (3.5)
** T3 (*n*, %)**			9 (10.5)
** T4 (*n*, %)**			5 (5.8)
** Nx (*n*, %)**			2 (2.3)
** N0 (*n*, %)**			6 (7.0)
** N1 (*n*, %)**			10 (11.6)
** Mx (*n*, %)**			1 (1.2)
** M0 (*n*, %)**			9 (10.5)
** M1 (*n*, %)**			8 (9.3)
**Physicians**			
** Sociodemographics**			
** Female gender (*n*, %)**			14 (63.3)
** Age in years, mean (SD)**			52
** Years of experience, median (range)**			13 (5-25)
** Communication skills training during:**			
** Medical school, yes (*n*, %)**			0 (0)
** Residency, yes (*n*, %)**			2 (7.7)
** Post-education, yes (*n*, %)**			4 (23.1)
** Type of hospital**			
** Academic (*n*, %)**			6 (46.2)
** Non-academic (*n*, %)**			7 (53.8)

#### Primary outcome

The primary outcome was observed SDM as assessed from the audio-recorded consultations using the Observing Patient Involvement in Decision-Making (OPTION5) instrument,^19^ with 5 items: (1) Are multiple options presented?, (2) Is a partnership with the patient established?, (3) Are the options described?, (4) Is the patient asked for their preferences?, (5) Are patients’ preferences included in the decision about next steps? Items are scored from 1 (no effort to involve the patient) to 4 (exemplary effort to involve the patient). The sum of these items was rescaled from 0 to 100.^20^ Two independent and experienced assessors, blinded to the research questions and randomization assignment, independently rated the recordings, applying the OPTION5 coding scheme.^19^ These assessors started double coding a set of ten audio recordings, and resulting scores were discussed. Inter rater agreement was calculated after each set of ten and considered sufficient if the Intraclass Correlation was higher than .60, reflecting substantial agreement.^17^ When the Inter Rater agreement was insufficient, this process was repeated. After the first set of ten consultations, the Inter Rater agreement was calculated. The Intraclass correlation turned out to be sufficient and strong (.81). The remaining recordings were scored independently by two raters without further discussions. Because the study focused on the general effect of PtDAs rather than a single decision-specific tool, we deliberately used a composite outcome, and the study was powered accordingly.

#### Secondary outcomes

##### Patient-reported SDM

Patients gave their evaluation of the SDM process using CollaboRATE, 3-items, ranging from 0 (no effort) to 9 (all possible effort) addressing explanations, preference elicitation, and preference integration.^21^

##### Well-being and worries

Patients rated their *general health* during the last week on an 11-point scale ranging from 0 (very bad) to 10 (excellent).

C*ancer worries* were scored with three questions,^22^^,^^23^ on a 4-point scale ranging from 1 (not at all) to 4 (always). *Trust in physician* was measured with the Wake-Forest Trust in Physician questionnaire, ten items scored on a 5-point scale, ranging from 1 (totally disagree) to 5 (totally agree).^24^

##### Information-related outcomes

*Subjective knowledge* patients rated their own knowledge about DTC using three questions, on a 10-point scale ranging from 1 (very bad) to 10 (excellent), which were subsequently averaged.

*Objective knowledge* on DTC was measured with five statements (right/wrong), generated by independent experts.

Two additional questions were asked: ‘having received any *undesired information* (yes/no)’ and whether ‘treatment options were presented in a *balanced way* (yes/no)’.

##### Decision-related outcomes

*Strength of treatment preference* was asked on a 5-point scale ranging from 0 (no preference) to 4 (very strong preference).

*The Decision Evaluation Scales* measures patient experiences with the decision using three scales, namely satisfaction-uncertainty, informed choice, and decision control,^25^ and measured on a 5-point scale, ranging from 1 (totally disagree) to 5 (totally agree). Having weighed the pros and cons was assessed by asking about three items, measured on a 5-point scale, ranging from 1 (totally disagree) to 5 (totally agree).^26^

*Preference for decision-making* Perceived participation in decision making was measured with two decision-making items from the Problem-Solving Decision-Making Scale (PSDM).^27^ These were measured on a 5-point scale ranging from 1 (doctor alone) to 5 (I alone). *Perceived involvement* was assessed after the decision using the same questions in the past tense. Patients were also asked whether they felt they had a choice (yes/no), and whether their opinion regarding the treatment mattered (yes/no).^28^

##### Values agreement

Patients values were asked with two questions weighing QOL against survival.^29^ Measured on a 5-item scale ranging from 1 (not agree) to 5 (totally agree). The items were: ‘If a treatment can prolong my life, I will always accept it, whatever the side effects may be’, and ‘If a treatment that prolongs my life results in such complications that I am prevented from leading a normal life, I would not have it’. Physicians answered two identical questions but now preceded by: ‘Which preference does the patient have?’.

### Statistical analysis

Descriptive statistics as percentages or means (with standard deviation; SD) were used. Differences between OPTION5 scores between the PtDA and usual care (UC) group were tested using *t*-tests. All remaining tests were descriptive and done with *t*-tests for independent samples; if a baseline measure was available, analysis of covariance (ANCOVA) were conducted. The level of values agreement between physician and patient was analyzed using Cohen’s kappa. Patient and physician scales were dichotomized. Tests were considered statistically significant if *P* < .05. We used SPSS Statistics v. 27.0 (IBM, Armonk, NU USA).

### Sample size

The effect size for improvement of the OPTION5 score was estimated to be 1. This effect size was reduced to 0.64 to account for (1) contamination as each physician sees both intervention and control patients, and (2) the effect of the communication training which may elevate OPTION5 scores in both arms, thus reducing possible differences. To detect an effect size of 0.64, with a two-sided test and *α* = .05, and a power of 0.80, a total of 78 patients are needed.

## Results

### Participants

In total 86 consecutive patients were included: 44 (51.2%) were randomized in the PtDA group and 42 (48.8%) in the UC group (Figure 1). Fourteen physicians included patients in the trial. Patient and physician characteristics are shown in Table 1.

Audio-recordings of consultations were collected for 78 (90.7%) patients, 73 (84.9%) patients returned the T2 questionnaire. Physicians’ questionnaires were received for 65 (75.6%) consultations.

### Primary outcome: observed SDM

The intervention had no effect on observed SDM as measured with the OPTION5 (Table 2). Of the five OPTION items, item 2 (establish a partnership) scored lowest in the UC group and item 5 (preferences included in the decision) scored lowest in the PtDA group. Item 1 (present multiple options) the highest in both groups. Differences between the PtDA and UC groups for the five OPTION items separately were also not statistically significant. Mean OPTION5 scores were respectively 52 (range 25-90, PtDA group) and 54 (range 15-90, UC group).

**Table 2.** Mean observer OPTION5 scores between the PtDA and usual care group, scaled to a score out of 100.

Short description of items	PtDA group (*n* = 38)	Usual care group (*n* = 40)
Item 1: Does the clinician present multiple options?*	14.2 (2.8)	14.2 (2.8)
Item 2: Does the clinician establish a partnership with the patient?*	7.9 (3.6)	7 (5.2)
Item 3: Are the options described?*	11.6 (3.2)	11.7 (3.6)
Item 4: Does the clinician ask the patient for their preferences?*	10.9 (3.6)	12.4 (4.0)
Item 5: Are the patients’ preferences included in the decision about next steps?*	7.7 (3.2)	8.8 (4.0)
OPTION5**, mean (SD)	52 (14.5)	54 (13.6)

* Range 0-20.

** Range 0-100.

### Secondary outcomes

No differences were found between the PtDA and UC group for the CollaboRATE scores, general health, cancer worries, and trust in the physician (Table 3).

**Table 3.** Unadjusted mean scores (SD) and results from the ANCOVA comparing the PtDA and usual care group.

	Group	T1	T2	F	*P*
**Well-being^a^**					
** General health**	PtDA (*n* = 36)	6.83 (1.63)	6.92 (1.30)	0.030	0.86
	Usual care (*n* = 33)	6.67 (1.34)	6.88 (0.96)		
** Cancer worries**	PtDA (*n* = 36)	2.25 (0.65)	2.04 (0.61)	2.9	0.09
	Usual care (*n* = 32)	2.10 (0.60)	2.16 (0.57)		
** Trust in physician**	PtDA (*n* = 36)		4.44 (0.59)	0.64	0.92
	Usual care (*n* = 32)		4.43 (0.51)		
**Information related^a^**					
** Information unpleasant**	PtDA (*n* = 36)	1.92 (0.28)	1.86 (0.35)	0.07	0.80
	Usual care (*n* = 32)	1.94 (0.25)	1.84 (0.37)		
** Information balanced**	PtDA (*n* = 38)		3.11 (0.76)	0.69	0.25
	Usual care (*n* = 35)		2.91 (0.61)		
** Subjective knowledge**	PtDA (*n* = 33)	6.10 (1.37)	6.81 (0.95)	0.004	0.95
	Usual care (*n* = 27)	6.30 (1.15)	6.87 (1.01)		
** Objective knowledge**	PtDA (*n* = 38)		4.0 (0.64)	1.5	0.99
	Usual care (*n* = 35)		4.0 (0.79)		
**Decision related^a^**					
** Decision satisfaction-uncertainty**	PtDA (*n* = 38)		4.1 (0.57)	0.20	0.20
	Usual care (*n* = 35)		3.9 (0.59)		
** Informed choice**	PtDA (*n* = 38)		4.1 (0.61)	0.008	0.17
	Usual care (*n* = 35)		3.9 (0.60)		
** Decision control**	PtDA (*n* = 38)		4.2 (0.64)	0.063	0.45
	Usual care (*n* = 35)		4.1 (0.68)		
** Weighing pros and cons**	PtDA (*n* = 38)		3.78 (0.59)	1.43	0.55
	Usual care (*n* = 35)		3.86 (0.47)		
** Treatment preference**	PtDA (*n* = 36)	1.50 (0.81)	1.25 (0.55)	1.38	0.24
	Usual care (*n* = 32)	1.66 (0.83)	1.44 (0.67)		
** Strength of treatment preference**	PtDA (*n* = 36)	2.33 (1.43)	3.03 (1.16)	0.61	0.44
	Usual care (*n* = 32)	2.13 (1.34)	2.78 (1.21)		
** Values**	PtDA (*n* = 36)	3.33 (0.51)	3.60 (0.83)	1.82	0.18
	Usual care (*n* = 32)	3.60 (0.69)	3.61 (0.67)		
** Preference for decision making**	PtDA (*n* = 36)	3.43 (0.74)	3.60 (0.83)	0.01	0.91
	Usual care (*n* = 32)	3.42 (0.76)	3.61 (0.67)		
** Perceived involvement**	PtDA (*n* = 36)		1.07 (0.21)	1.12	0.62
	Usual care (*n* = 32)		1.05 (0.15)		
** CollaboRATE**	PtDA (*n* = 20)		7.60 (0.48)	0.005	0.85
	Usual care (*n* = 20)		7.57 (0.60)		

Abbreviations: *SD,* standard deviation; ANCOVA, analysis of covariance; PtDA, Patient Decision Aid.

* *P* < .05.

^a^ More information on the scales can be found in Table 1.

No differences were found for objective and subjective knowledge, receiving undesired information and balanced presentation of the information (Table 3).

No differences were found in strength of treatment preference, decision satisfaction-uncertainty, informed choice and decision control between the two groups (Table 3). At follow-up (T2), no differences were found regarding treatment preferences. Most patients reported having a treatment preference (91.8%); out of these, most (61.2%) favored the more conservative option. There was no values agreement between patients and physicians about relative importance of the values QOL and survival (Table 4).

**Table 4:** Agreement between patients and physicians about the importance of values.

	Physicians	Not agree *n*(%)	Agree *n*(%)	Total *n*(%)	Cohen’s kappa
	Question 1 ‘If a treatment can prolong my life, I will always accept it, whatever the side effects may be’				
	Not agree	27(47)	9(16)	36(63)	
	Agree	9(16)	12(21)	21(37)	
**Patients**	Total	36(63)	21(37)	57(100)	0.321*
	Question 2 ‘If a treatment that prolongs my life results in such complications that I am prevented from leading a normal life, I would not have it’				
	Not agree	7(12)	14(25)	21(37)	
	Agree	11(19)	25(44)	36(63)	
	Total	18(32)	39(68)	57(100)	0.029

* *P *< .05.

The PtDA was evaluated with Beta testing during the trial (Table 5) by thirty-three (75%) out of 44 patients in the PtDA group. Twenty-seven (81.8%) said they used the PtDA and 21 of these (77.8%) filled out the values clarification exercise and thought it was helpful in the decision making (Table 5). Twenty-two (81.5%) patients mentioned the PtDA improved their knowledge about DTC, length and amount of information was considered right by 23 (85.2%) and 25 (92.6%) patients, respectively. Comprehensibility (25, 92.6%) and credibility (27, 100%) were good. The overall grade was 7.7. All patients indicated that they would recommend the PtDA.

**Table 5.** Results of Bѐta testing questionnaire.

Bèta testing *n* %	44 (100)
**Filled out questionnaire**	33 (75)
**Used PtDA**	27 (81.8)
**Filled out values clarification exercise**	21 (77.8)
**Values clarification exercise helpful in decision making**	21 (77.8)
**Improved knowledge**	22 (81.5)
**Length just right**	23 (85.2)
**Amount of information just right**	25 (92.6)
**Comprehensibility good**	25 (92.6)
**Credibility good**	27 (100)
**Recommend PtDA**	27 (100)
**Time burden (range in minutes, mean)**	10-90 (39)
**Average grade (range 0-10)**	7.7

## Discussion

To our knowledge, this is the first RCT to evaluate the impact of a PtDA on observed SDM in patients with low-risk and advanced RAI refractory DTC. The PtDAs were evaluated for effectiveness on SDM while all physicians received a SDM training. In contrast to our hypothesis, the PtDAs did not elevate observed SDM in this setting. Secondary patient reported outcomes were also not affected. Nevertheless, patients mentioned that the PtDA was readable and helpful for decision making and they recommended using the PtDA.

Regarding other PtDAs in low-risk DTC patients (1-4 cm), there is only one published PtDA focused on RAI therapy.^30^ This PtDA is limited to the decision to follow or omit RAI treatment after TT. On the other hand Brito et al^31^ and Pitt et al^32^^,^^33^ developed a treatment choice tool (paper cards) for patients with DTC. These tools included the option of active surveillance, implying that these tools are also useful for informed patients with mPTC. More recently, O’Neill et al^34^ developed a decision aid for patients with Bethesda 3-6 thyroid nodules for de-escalation of surgery. All these tools need further testing a RCT.

The mean OPTION5 scores of 52-54 (full-scale of 0-100), in both groups are relatively high. In contrast, lower OPTION5 scores of 23 were found in patient groups with cancer, diabetes and depression^35^; scores ranging from 16.8 to 28.3 were reported among untrained specialists in the Netherlands from 18 different disciplines.^36–38^ Thus, the training yielded a high level of OPTION5 scores of the trained physicians in our study.^39^ In line with this, a training, together with a PtDA, resulted in higher mean OPTION5 scores of 37.8 in patients undergoing vascular surgery.^40^ Likewise, higher scores of about 50-54 were reported in physicians using an option grid for osteoarthritis of the knee,^41^ or when a multilevel implementation program was used in breast cancer patients including a communication training without a PtDA.^42^ High OPTION5 scores may further be explained by participation and motivation bias as only half of the approached hospitals consented to participate.

Importantly, there is no consensus about the level of an acceptable OPTION5 score. The manual of the Observer OPTION5 suggest that a sum score of ≥75 (0-100) represents a skilled effort, involving substantive phrases or sentences.^20^ Overall, despite the relatively high OPTION5 scores in our study, there was no values agreement between patients and physicians, indicating that physicians could not estimate to what extent treatment values were important to patients. In our study, only eight conversations achieved a sum score of ≥75, with a tendency to improve values agreement. However, physicians reaching these scores were more focused on item 3 (describing the options) and 4 (preference elicitation) with still only minimal values agreement. It remains thus unclear whether and how values agreement can be improved. Thereby, based on the data from this study, we cannot establish whether the outcome would have been different if the study would have focused only on one patient population, for example on advanced DTC or low-risk DTC. A subsequent study focusing on the additive value of PtDAs in the different patient populations, potentially having different informational needs and decision preferences depending on the decision they face and depending on the phase of the disease, is warranted.

The high OPTION5 scores in our study, may have resulted in a ceiling effect precluding additional improvement in the intervention group. This is in line with Henselmans et al^14^ reporting OPTION5 scores of 50 in trained physicians without further improvements after adding a communication aid in a palliative setting. This ceiling effect could also explain why in contrast to previous studies,^13^^,^^43^ no positive effects on the secondary outcomes were found. DTC specific factors could have affected the results as well. Patients with advanced DTC are more familiar with the disease given its lengthy course, explaining the lack of improvement of knowledge with the PtDA. Furthermore, the low level of cancer worries in both groups suggests that there is little room for improvement regardless the use of a PtDA. A recent study also showed that regardless of chosen treatment decision (TL or TT), patients with low-risk DTC reported low levels of DTC related fear and worries.^44^

Despite the lack of improvements on primary and secondary outcomes, most patients appreciated the PtDA, highlighting the importance of its future routine implementation. However, the current evidence indicates that the implementation of PtDAs remains challenging.^45^ Often SDM consultations take place directly after sharing the cancer diagnosis, which may find the patient unprepared for participation in the decision making, thus optimal timing of the PtDA is a main logistic barrier.^46^ In our study, some patients felt they did not have the time to use the PtDA sufficiently while others may have received additional sufficient information already before the SDM consultation from their referring physician, limiting the utility of the PtDA. Having access to the PtDA, before the SDM consultation, ensuring sufficient time to examine it and ensuring comprehensibility for patients with limited health literacy remain essential.^47^ In addition, training physicians in shared decision-making skills should be prioritized. Although various training options exist, including online programs, their optimal effectiveness remains to be studied.

## Strengths and limitations

Apart from its prospective randomized trial design, the study is strengthened by the contribution of physicians from different backgrounds, patients, and patient advocates in designing and assessing the PtDAs. The focus on patients’ values in the communication training for physicians and in the values clarification exercise present in the PtDA for patients, makes this study unique in its potential to improve values agreement between physicians and patients. Furthermore, the execution of the study in the context of direct clinical environment despite differences in logistics between hospitals, makes the results more relevant for current practice.

Some limitations must be acknowledged. First, we trained all physicians participating in the study, resulting in the lack of a control group without communication training. Second two types of patient groups and different physician specialties were included possibly increasing variability. However, this is also a positive characteristic as it reflects clinical practice. Third, despite our efforts to blind the physicians, the recordings indicate that use of the PtDA was noticed by them, potentially affecting the physicians’ behavior and quality of SDM. Fourth, as no OPTION5 scores were obtained before the training of physicians, the effect of the training cannot be assessed. Lastly, generalizability is limited as the study likely underestimates the effect of a PtDA in this trained population, because in current clinical practice most physicians are untrained.

## Conclusion

Mean OPTION5 scores were high compared to other trials but the PtDA did not lead to higher SDM and patient outcomes while all physicians were trained in SDM. Nevertheless, all patients recommended the PtDA. Therefore, given positive results of PtDAs in the literature in untrained physicians, we consider, our PtDAs are useful to support physicians and patients in decision making,^13^ because usually training is not available. Future research should focus on ways to improve SDM.

## Supplementary Material

oyag126_Supplementary_Data

## Data Availability

The data that support the findings of this study are available on request from the corresponding author. The data are not publicly available due to privacy or ethical restrictions.
