# Supplementary material for: Effect of a patient decision aid on shared decision making in patients with differentiated thyroid cancer: a randomized controlled trial
Source: Oncologist. 2026 Apr 7;31(5):oyag126. doi: 10.1093/oncolo/oyag126 (PMC13129192; doi:10.1093/oncolo/oyag126)
Supplement: oyag126_Supplementary_Data [file oyag126_supplementary_data.zip › Supplementary Table 1.docx]

**Supplementary Table 1.** Overview of outcome measures

Measures Operationalisation t1 t2

Primary outcome measure

Quality of SDM OPTION-5 score from audio-recording

Secondary outcome measures t1 t2

Information related outcomes

Subjective knowledge 1-10 (extremely poor – excellent) X X

Objective knowledge Five statements judged as right or wrong X

Perceived participation Problem-Solving Decision Making Scale X

Perceived involvement Yes/no X

Information unpleasant Yes/no X X

Information balanced Yes/no X

Decision-related outcomes

Decision satisfaction-uncertainty Decision Evaluation Scales X

Informed choice Decision Evaluation Scales X

Decision control Decision Evaluation Scales X

Weighing pros and cons X

Number of treatment options X X

Strength of treatment preference 1-4 (not strong-very strong) X X

Values Importance ratings X X

Well-being

Cancer worries Adapted Lerman’s Cancer Worry Scale X X

Trust Wake-Forest Trust in Physician X

General health 1-10 (very bad health – excellent health) X X

Communication

Subjective evaluation of CollaboRATE X

SDM process

Duration of the consultation Minutes from audio-recording

Treatment choice been made Yes/no from audio-recording

Additional consult Yes/no from audio-recording
