# Supplementary material for: Effect of a patient decision aid on shared decision making in patients with differentiated thyroid cancer: a randomized controlled trial
Source: Oncologist. 2026 Apr 7;31(5):oyag126. doi: 10.1093/oncolo/oyag126 (PMC13129192; doi:10.1093/oncolo/oyag126)
Supplement: oyag126_Supplementary_Data [file oyag126_supplementary_data.zip › Supplemental Dataset.docx]

**Supplemental Dataset.** Hyperlinks of the PtDAs in Dutch and translated PtDAs in English

[Operatie van de schildklier bij schildklierkanker NL](https://schildklier.keuzehulp.net/)

[Sorafenib: Wachten of starten met systeemtherapie bij uitgebreide schildklierkanker NL](http://sorafenib.keuzehulp.net/)

[Lenvatinib: Wachten of starten met systeemtherapie bij uitgebreide schildklierkanker NL](https://lenvatinib.keuzehulp.net/)

Decision Aid

Removal of half or the entire thyroid gland in thyroid cancer

This decision aid is for patients with thyroid cancer who are facing the choice between removal of half of the thyroid gland or removal of the entire thyroid gland.

This decision aid discusses the advantages and disadvantages of both options. It can help you prepare for your appointment with your doctor or nurse. Together, you will choose the treatment that best suits you.

You will be guided through the decision aid in 6 steps.

You can print an overview of your considerations and notes. This allows you to review everything calmly and discuss it with your family or friends.

**Step 1: Introduction**

**The thyroid gland**

The thyroid gland is located at the front of the neck, next to the windpipe. It consists of two lobes. These lobes are made up of small sacs called follicles. The thyroid produces two types of thyroid hormone. These hormones regulate metabolism and growth.


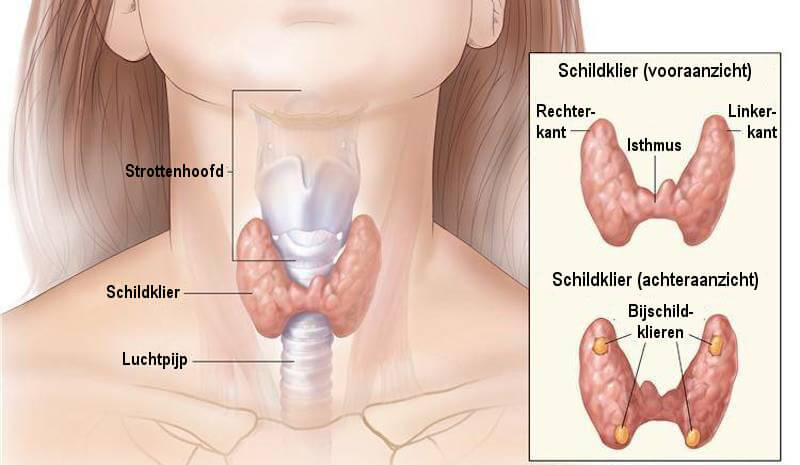


**What is thyroid cancer?**

Thyroid cancer is a malignant tumor of the thyroid gland. There are several types of thyroid cancer. Most patients have the *papillary* or *follicular* type. These tumors usually grow slowly and can often be cured with surgery.

Each year, approximately 900 people in the Netherlands are diagnosed with thyroid cancer.

More information about thyroid cancer can be found at:
[www.schildklier.nl](http://www.schildklier.nl)
[www.kanker.nl](http://www.kanker.nl)

**Step 2: Treatments**

**What are the treatment options?**

The aim of surgery is to remove the cancer cells. There are two options:

- Surgery in which half of the thyroid gland is removed
- Surgery in which the entire thyroid gland is removed

Both options have advantages and disadvantages.

Recent research increasingly shows that removal of half of the thyroid gland is a comparable treatment option and does not affect prognosis.

**Removal of half of the thyroid gland**

Removal of half of the thyroid gland usually takes 1 to 1.5 hours. You will be under general anesthesia. The surgeon makes a horizontal incision low in the neck, allowing removal of half of the thyroid gland.

Pain after surgery is usually mild. It is comparable to a sore throat and disappears within a few days. The wound heals quickly and the stitches dissolve on their own.

If half of your thyroid gland is removed, the remaining half will usually take over hormone production. About 12 out of 100 people need to start taking thyroid hormone tablets after surgery. During hospital follow-up visits, your thyroid function will be checked with blood tests. An ultrasound of the neck will also be performed.

Because you retain one half of the thyroid gland, you cannot receive additional radioactive iodine treatment.

If the tumor turns out to be larger or more aggressive than expected after surgery, there is a chance that a second operation will be required to remove the remaining half of the thyroid gland.

**Complications after removal of half of the thyroid gland**

Every surgery carries risks. Thyroid surgery also involves a risk of general complications such as bleeding or wound infection. In addition, the following complication may occur:

- Damage to one of the vocal cord nerves, which may cause a change in your voice.

**Advantages of removing half of the thyroid gland:**

- You keep one half of your thyroid gland
- You will probably not need to take thyroid hormone tablets for the rest of your life
- Lower risk (<2%) of damage to the vocal cord nerve with permanent voice changes
- Shorter hospital stay compared to removal of the entire thyroid gland

**Disadvantages of removing half of the thyroid gland:**

- You cannot receive radioactive iodine treatment
- There is a chance that a second surgery will be needed
- You will probably be monitored for a longer period, with more frequent neck ultrasounds

**Removal of the entire thyroid gland**

Removal of the entire thyroid gland usually takes 1 to 2 hours. You will be under general anesthesia. The surgeon makes a horizontal incision low in the neck, allowing removal of the entire thyroid gland, similar to partial thyroid removal.

Pain after surgery is usually mild and comparable to a sore throat. It disappears within a few days. The wound heals quickly and the stitches dissolve on their own.

**Thyroid hormone**

If your entire thyroid gland is removed, your body no longer produces thyroid hormone. From that moment on, you will need to take tablets containing the thyroid hormone levothyroxine. You will receive these tablets after surgery.

It may take some time to determine the correct dosage. During this adjustment period, you may experience fatigue, restlessness, or low energy. These symptoms may also persist.

**Radioactive iodine**

Four to six weeks after surgery, you may receive radioactive iodine-131 treatment in consultation with your treating physician. The aim is to destroy any remaining (malignant) thyroid cells.

**Follow-up**

Approximately 12 months after radioactive iodine treatment, an ultrasound of the neck will be performed. In addition, thyroglobulin (Tg) will be measured in your blood. Tg is a protein produced only by thyroid cells or thyroid cancer cells and is known as a tumor marker.

Ultrasound and Tg measurements are used to assess whether the treatment was successful and whether cancer cells are still present.

More information about Tg can be found at:
[www.schildklier.nl](http://www.schildklier.nl)

**Complications after removal of the entire thyroid gland**

Every operation carries risks. Thyroid surgery also involves a risk of general complications such as bleeding or wound infection. In addition, two other complications may occur:

1. Damage to one or both vocal cord nerves, which may cause voice changes
2. Loss of the parathyroid glands, resulting in low calcium levels in the blood

**Advantages of removing the entire thyroid gland:**

- Radioactive iodine can be given to destroy remaining thyroid and thyroid cancer cells
- The tumor marker Tg can be used to monitor recurrence

**Disadvantages of removing the entire thyroid gland:**

- The operation is more extensive and recovery takes longer
- You must take thyroid hormone tablets for life; some people experience persistent symptoms
- The risk of complications is twice as high compared to partial removal, including damage to the vocal cord nerves (5%) causing permanent voice changes or loss of the parathyroid glands (4%), requiring calcium supplements

**Results**

Below are the results of the two surgical options. We look at the risk of thyroid cancer recurrence and survival after 10 years.


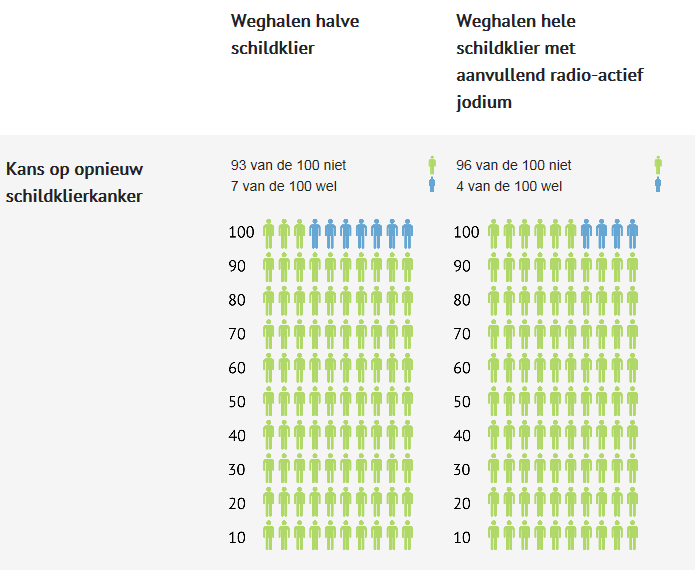
**
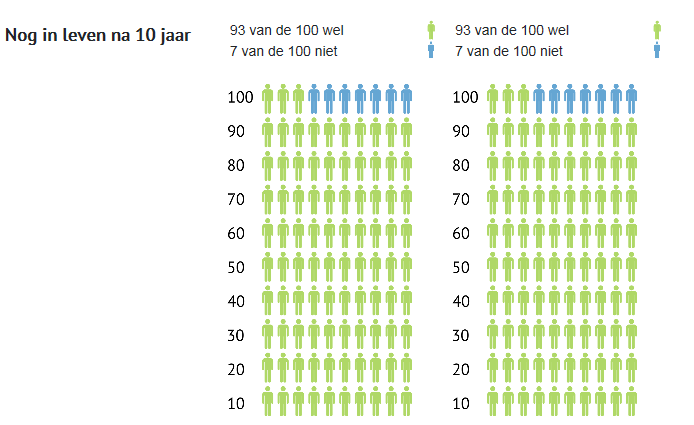
**

**Step 3: Compare the treatments**

|  | **Removal of half of the thyroid gland** | **Removal of the entire thyroid gland with additional radioactive iodine** |
| --- | --- | --- |
| **What is it?** | Surgery under general anesthesia in which half of the thyroid gland is removed. | Surgery under general anesthesia in which the entire thyroid gland is removed. |
| **Use of medication?** | Probably no thyroid hormone tablets needed. | Daily, lifelong thyroid hormone tablets required. |
| **What are the possible complications?** | <2% risk of damage to the vocal cord nerve, resulting in permanent voice changes. | 5% risk of damage to the vocal cord nerves, resulting in permanent voice changes.  4% risk of loss of the parathyroid glands, requiring calcium tablets. |
| **How long does the treatment take?** | Surgery: 1 to 1.5 hours  Hospital stay: 1 day | Surgery: 1 to 2 hours  Hospital stay: 2 days |
| **How effective is the treatment?** | Thyroid cancer returns in 7 out of 100 patients. | Thyroid cancer returns in 4 out of 100 patients. |
| **Can radioactive iodine be given?** | Radioactive iodine treatment is not possible. | Additional radioactive iodine treatment can be given. |
| **Is there a difference in survival?** | Survival after 10 years is the same. | Survival after 10 years is the same. |

**Step 4: What you need to know**

These questions relate to what you have read in the decision aid. You can always review the information again or discuss it with your doctor or nurse practitioner.

1. **If I have my entire thyroid removed, I must take thyroid hormone tablets for the rest of my life.**
   True / False
2. **The chance that my tumor will return is higher if only half of my thyroid is removed.**
   True / False
3. **If I have half of my thyroid removed, I have a lower risk of complications.**
   True / False
4. **If I have my entire thyroid removed, I will live longer.**
   True / False
5. **If I have my entire thyroid removed, I can be treated with radioactive iodine.**
   True / False
6. **If I have half of my thyroid removed, the marker for tumor recurrence, thyroglobulin (Tg), can be used.**
   True / False

Correct answers:

1. True. If the entire thyroid is removed, your body no longer produces thyroid hormone and you must take thyroid hormone tablets.
2. True. Cancer returns in 7 out of 100 people after removal of half of the thyroid gland, compared to 4 out of 100 after removal of the entire thyroid gland.
3. True. The risk of complications is half as high.
4. False. Ten-year survival is the same for both surgeries.
5. True. After complete removal of the thyroid gland, radioactive iodine can destroy remaining (malignant) thyroid cells.
6. False. Tg is only used after complete thyroid removal and radioactive iodine treatment as a marker to monitor recurrence.

**Step 5: What is important to you**

You can choose between removal of half or the entire thyroid gland. Together with your doctor, you decide which option suits you best, depending on what matters most to you.

Your doctor invites you to bring your answers on this and the following pages to your next appointment.

1. **If I have my entire thyroid gland removed, I will need to take thyroid hormone tablets for the rest of my life. I find this…**

Not important Slightly important Important Very important

1. **Adjusting the dose of thyroid hormone tablets may reduce my quality of life. I find this…**

Not important Slightly important Important Very important

1. **If I have half of my thyroid gland removed, I may be able to go home the same day. I find this…**

Not important Slightly important Important Very important

1. **If I have my entire thyroid gland removed, I have twice the risk of (temporary) complications. I find this…**

Not important Slightly important Important Very important

1. **If I have my entire thyroid gland removed, I can receive additional treatment with radioactive iodine. I find this…**

Not important Slightly important Important Very important

1. **The risk that my tumor will return is slightly lower if the entire thyroid gland is removed with additional radioactive iodine treatment. I find this…**

Not important Slightly important Important Very important

Your doctor or nurse can think along with you better if he or she knows what is important in your life, for example your family, your work, or your hobbies. You can fill this in below.

*The following things are important in my life:*

Your doctor or nurse can also think along with you better if he or she knows what you are most concerned about. You can fill this in below.

*I am most concerned about:*

**My choice**
You have thought about what is important to you. Which treatment do you prefer at this moment?

- Removal of half of the thyroid gland
- Removal of the entire thyroid gland
- Do not know

**How strong is this preference?**

Not strong Fairly strong Strong Very strong

**Decision Aid**

Waiting or Starting Systemic Therapy for Advanced Thyroid Cancer

This decision aid is for patients with advanced thyroid cancer who are faced with the choice of waiting or starting treatment with systemic therapy.

In this decision aid, the advantages and disadvantages of waiting or starting systemic therapy are discussed. It can help you prepare for your appointment with your doctor or nurse. Together, you can choose the treatment that best suits you.

The decision aid guides you through six steps. You can start by clicking the Start Decision Aid button. On each page, you can click to go to the next (or previous) page.

You can print an overview of your considerations and notes. This allows you to review everything calmly and discuss it with your family or friends.

**Step 1: Introduction**

**The Thyroid**
The thyroid is a gland located at the front of the neck, against the windpipe. It consists of two lobes. These lobes are made up of small sacs called follicles. The thyroid produces two types of thyroid hormones, which regulate metabolism and growth.


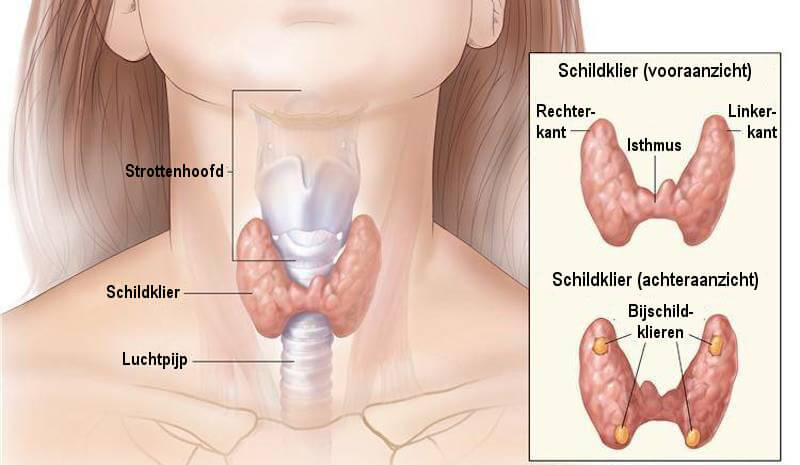


**What is metastatic thyroid cancer**

Thyroid cancer is a malignant tumor in the thyroid gland. There are several types of thyroid cancer. Most patients with thyroid cancer have the “papillary” or “follicular” type. If cancer cells remain in the body after surgery and treatment with radioactive iodine, this is called metastasis. Metastases are usually found in the lymph nodes, lungs, and/or bones, but other organs can also be affected, such as the liver and brain.

Each year, about 900 people in the Netherlands are diagnosed with thyroid cancer.

For more information about thyroid cancer, you can visit:
[www.schildklier.nl](https://www.schildklier.nl)
[www.kanker.nl](https://www.kanker.nl)

**Step 2: Treatments**

**What are the treatments?**
Do you have advanced thyroid cancer?
Then, after discussing with your doctor, you may consider systemic therapy.
You can choose between:

- Waiting to start systemic therapy until the tumor grows further or causes symptoms
- Starting systemic therapy immediately

Both options have advantages and disadvantages.

**What is Systemic Therapy**

Systemic therapy is a treatment with medication. These medicines are given in tablet form and spread through your entire body via the bloodstream. Systemic therapy aims to kill cancer cells in your body or slow down their growth. This can help keep the tumor stable for a longer period or make it smaller. Systemic therapy does not cure the cancer. Its effect is temporary, and eventually the tumor will grow again.

The medication used in your case belongs to the group of targeted therapies. Chemotherapy is not used for thyroid cancer.

In the Netherlands, two medications are available for systemic therapy: Sorafenib and Lenvatinib. Your doctor will decide which medicine is most suitable for you; you do not have a choice in this.

**Waiting to Start Systemic Therapy**

Waiting to start systemic therapy can be an option if the tumor remains stable or grows slowly, and causes few or no symptoms for a long time. You will continue taking thyroid hormone tablets.

If you choose to wait, you will still need to visit the hospital for check-ups. This includes blood tests and scans of the tumor.

Your doctor will discuss with you how often you should come to the hospital for check-ups and scans.

**Advantages of waiting to start systemic therapy:**

- You do not need to take additional medication.
- You will visit the hospital less often for check-ups and scans.
- You will not experience side effects from the treatment.
- You can still start systemic therapy in the future if needed.

**Disadvantages of waiting to start systemic therapy:**

- The tumor may continue to grow.
- Tumor growth may lead to symptoms and could reduce your current quality of life.

**Starting Systemic Therapy**

You may start systemic therapy if:
the tumor continues to grow and causes symptoms, or is likely to cause symptoms soon.

During systemic therapy, you will be monitored regularly. This includes checking how the cancer responds to the treatment and whether you experience any side effects. You will continue taking thyroid hormone tablets.

Your doctor will discuss with you how often you need to come to the hospital for check-ups.

**Advantages of starting systemic therapy:**

- The tumor may remain stable for a longer period or shrink.
- You may experience fewer symptoms from a growing tumor.
- You receive medication that actively treats the cancer.

**Disadvantages of starting systemic therapy:**

- It is not clear whether patients using systemic therapy live longer.
- There is a high chance of side effects, some of which can be severe.
- Side effects may reduce your quality of life.
- At the start of treatment, you will need to visit the hospital more often for check-ups.

**Side Effects of Lenvatinib**

**Common side effects** **(affecting more than 30 out of 100 people) include:**

- Digestive issues, such as diarrhea, nausea, vomiting, loss of appetite, or constipation.
- Weight loss due to diarrhea or reduced appetite.
- Fatigue or headaches.
- High blood pressure. Your doctor will regularly monitor your blood pressure and may prescribe medication to lower it if necessary.
- Low platelet count, which increases the risk of bleeding. Contact your doctor if you notice bruises or nosebleeds.
- Painful mouth, tongue, or throat, dry mouth, hoarseness, and taste changes.
- Hand-foot problems. Your hands and feet may become red, painful, and swollen, or you may experience a burning sensation, cracks, blisters, calluses, or peeling. Inform your doctor if you experience these symptoms.

**Less common side effects** **(affecting 10 to 30 out of 100 people) include:**

- Dizziness, insomnia, or weakness.
- Urinary problems.
- Muscle and joint pain, back pain, or pain in the arms and legs.
- Skin rash. A rash may be caused by sensitivity but not always. Contact your doctor if a rash occurs.
- Hair changes. Your hair may become thinner, change color, or texture. After stopping Lenvatinib, hair usually starts growing back after about a month.
- Fluid retention.

With Lenvatinib, 97 out of 100 patients experience side effects. It is not possible to predict in advance which side effects a person will have or how severe they will be. What is mild for one person may be very bothersome for another. Side effects can also be temporary.

If you experience severe side effects, your doctor may reduce the dose of this medication. This happens in 68 out of 100 patients. Sometimes, treatment may be temporarily paused.

The average time the tumor remains stable or shrinks while using Lenvatinib is 10–18 months. Without Lenvatinib, this is on average 4 months.

**Results**


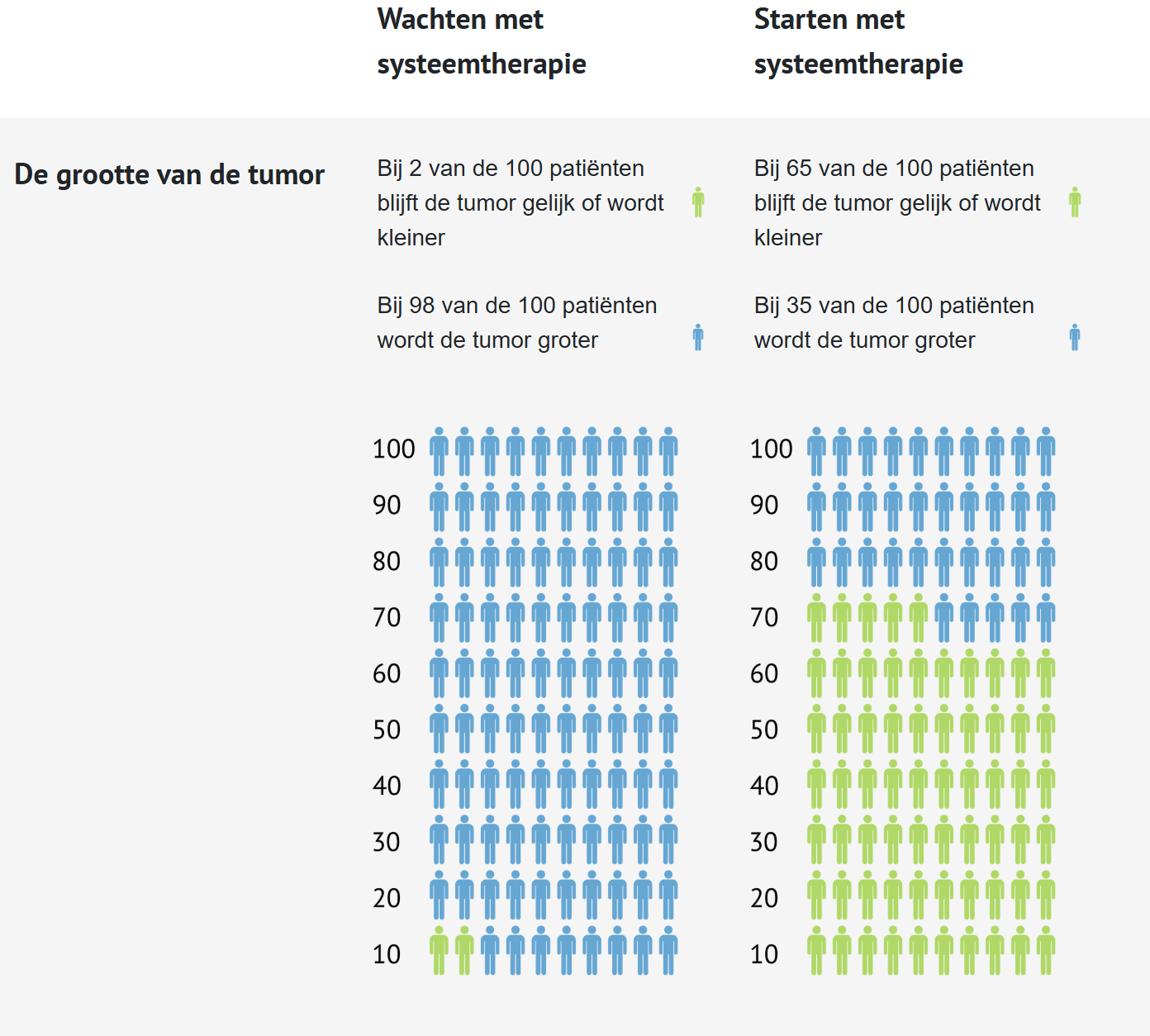


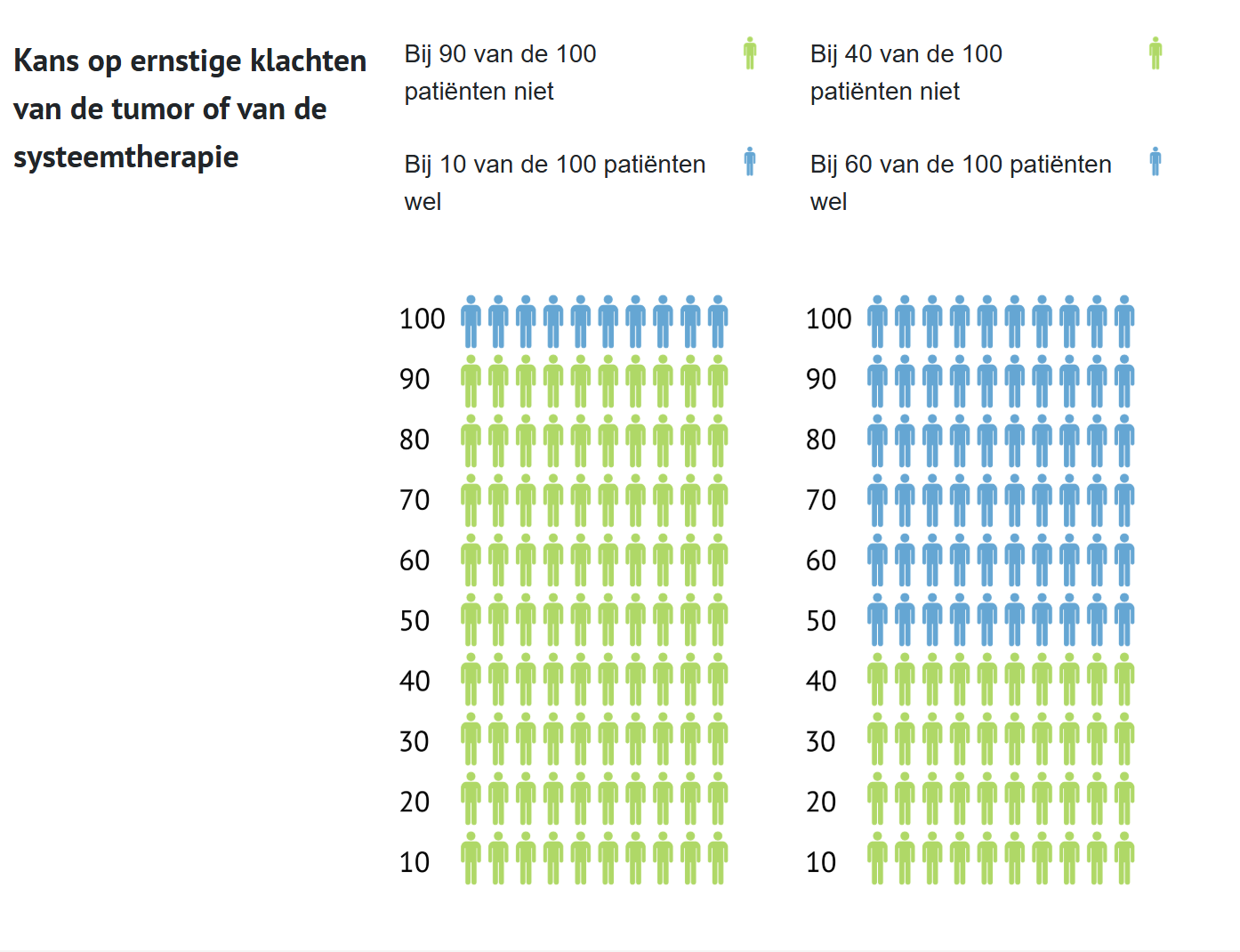


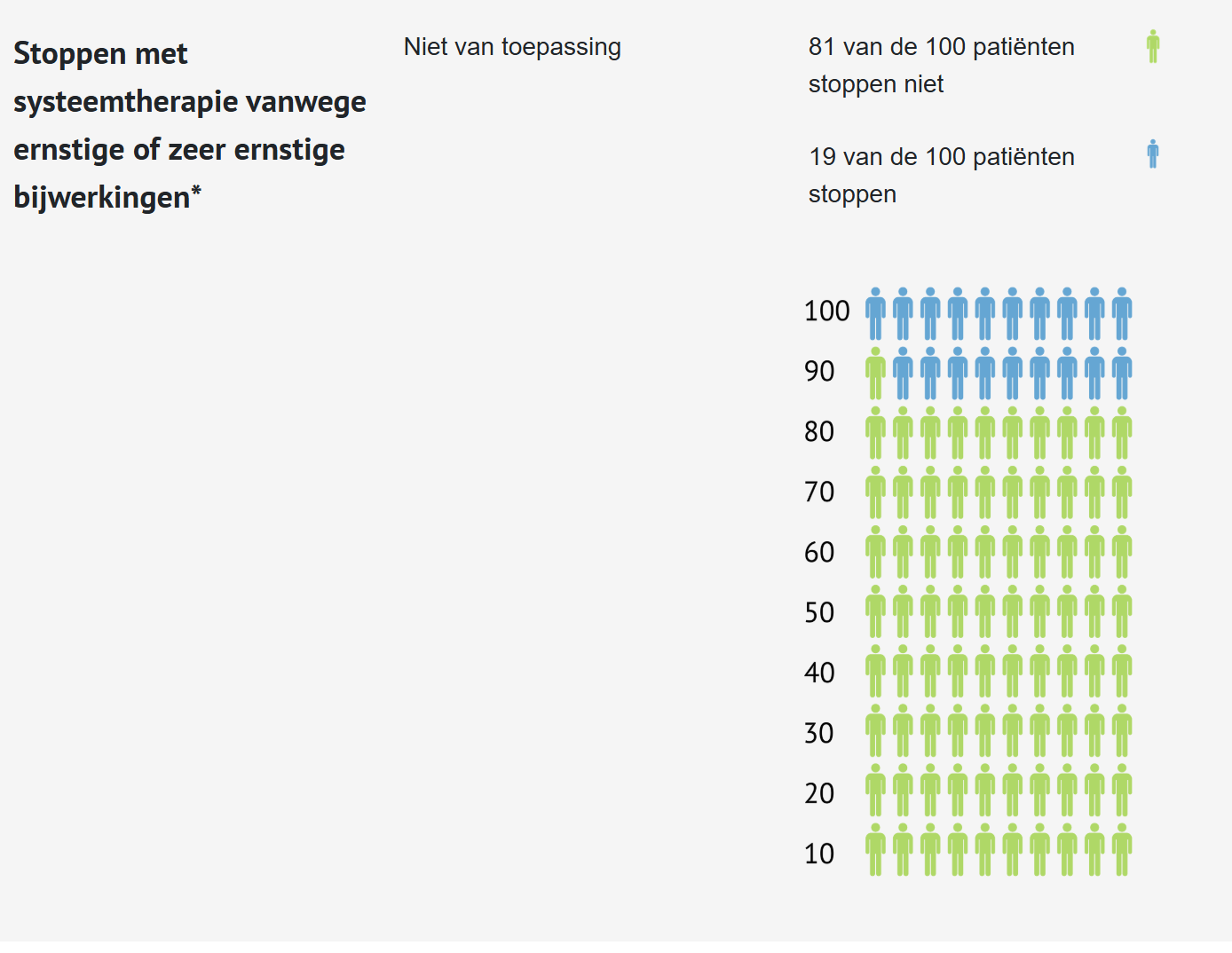


**Step 3** **Compare the treatments**

|  | **Waiting with systemic therapy** | **Starting systemic therapy** |
| --- | --- | --- |
| **What does it mean?** | No additional medications. Hospital visits for check-ups and scans. | Taking additional medications daily for a longer period. More frequent hospital visits for check-ups. |
| **What is the chance of side effects?** | No side effects from treatment. | High chance of side effects, which may reduce quality of life. |
| **How long does the treatment last?** | Observation until the tumor grows further or causes symptoms. | As long as the treatment affects the tumor and side effects are manageable. |
| **How effective is the treatment?** | The tumor may continue to grow. | The tumor may remain stable longer or become smaller, possibly reducing symptoms. |
| **How long can the tumor remain stable or become smaller?** | 4 months. | 10–18 months. |
| **Is there a difference in survival?** | At present, it is not clear whether patients live longer with systemic therapy. | At present, it is not clear whether patients receiving systemic therapy live longer. |

**Step 4: What you should know**

These questions are about what you have read in the decision aid. You can always review the information again or discuss it with your doctor or nurse specialist.

1. **Systemic therapy almost never causes serious side effects.**
   True / False
2. **If I wait to start systemic therapy, my quality of life will remain the same for now.**
   True / False
3. **If I start systemic therapy, my tumor may remain stable or shrink for a longer period.**
   True / False
4. **If I wait to start systemic therapy, symptoms caused by my tumor may get worse sooner.**
   True / False
5. **With systemic therapy, I do not know whether I will live longer.**
   True / False

**Correct answers:**

1. False. 37 out of 100 patients using Sorafenib experience serious side effects.
2. True. By waiting to start systemic therapy, you do not experience side effects from the treatment. Your daily functioning will remain the same for now.
3. True. In 54 out of 100 patients, the tumor shrinks or remains stable after starting systemic therapy.
4. True. By waiting to start systemic therapy, symptoms caused by the tumor may worsen sooner.
5. True. It is unclear whether systemic therapy prolongs life.

**Step 5: What is important to you?**

You can choose between waiting or starting systemic therapy. Together with your doctor, you will decide which option suits you best. This depends on what is important to you. For the statements below, you can indicate how important each one is to you.

Your doctor will invite you to bring your answers from this and the next page to your next appointment.

1. **If I wait to start systemic therapy, I will not have side effects, and my quality of life will remain the same for now.** **I find this…**

Not important A little important Important Very important

1. **If I start systemic therapy, my tumor may not grow for a longer period.** **I find this…**

Not important A little important Important Very important

1. **If I wait to start systemic therapy, I will need fewer hospital visits for check-ups.** **I find this…**

Not important A little important Important Very important

1. **If I start systemic therapy, I will have done everything I can about my disease.** **I find this…**

Not important A little important Important Very important

1. **If I wait to start systemic therapy, I can still start it later. I find this…**

Not important A little important Important Very important

1. **With systemic therapy, I do not know whether I will live longer. I find this…**

Not important A little important Important Very important

Your doctor or nurse can think along with you better if he or she knows what is important in your life, for example your family, your work, or your hobbies. You can fill this in below.

*The following things are important in my life:*

Your doctor or nurse can also think along with you better if he or she knows what you are most concerned about. You can fill this in below.

*I am most concerned about:*

**My choice**
You have thought about what is important to you. Which treatment do you prefer at this moment?

- Waiting to start systemic therapy
- Starting systemic therapy
- Don't know

**How strong is your preference above?**

Not strong Somewhat strong Strong Very strong
